# Theta burst stimulation for the acute treatment of major depressive disorder: A systematic review and meta-analysis

**DOI:** 10.1038/s41398-021-01441-4

**Published:** 2021-05-28

**Authors:** Jeffrey D. Voigt, Andrew F. Leuchter, Linda L. Carpenter

**Affiliations:** 1 99 Glenwood Road, Ridgewood, NJ USA; 2 University of California Los Angeles (UCLA), Neuromodulation Division, Semel Institute for Neuroscience and Human Behavior, Los Angeles, USA; 3 Brown University Department of Psychiatry and Human Behavior, Butler Hospital TMS Clinic and Neuromodulation Research Facility, Los Angeles, USA

**Keywords:** Depression, Scientific community

## Abstract

Patients with major depressive disorder (MDD) may be refractory to or have contraindications that preclude treatment with antidepressant pharmacotherapies. Alternative therapies such as repetitive transcranial magnetic stimulation (rTMS) continue to evolve, and include theta burst stimulation (TBS), which has advantages over conventional rTMS. The aim of this study was to identify and meta-analyze efficacy data from all randomized controlled trials (RCTs) investigating TBS as a treatment for MDD. Published reports of RCTs (January 1, 2010 to October 23, 2020) were identified via systematic searches in computerized databases, followed by review of individual reports for inclusion. Inclusion criteria included primary diagnosis of MDD ≥ 1 week duration of therapy with ≥10 sessions, and treatment with any form of TBS. The Cochrane GRADE methodology and PRISMA criteria were used for evaluation of individual trials. Data from ten RCTs were included, representing 667 patients. Of these, 8 RCTs compared TBS to sham treatment and one compared TBS to standard rTMS (i.e., high frequency stimulation over left dorsolateral prefrontal cortex [HFL]). Quality of evidence assessment yielded high confidence in the finding of TBS being superior to sham on response measured by the Hamilton Depression Rating Scale (HRSD) (RR = 2.4; 95% CI: 1.27 to 4.55; *P* = 0.007; *I*^*2*^ = 40%). Comparison of HRSD response rates for TBS versus rTMS produced no statistically significant difference (RR = 1.02; 95% CI: 0.85 to 1.23; *P* = 0.80; *I*^*2*^ = 0%). The incidence of adverse events between TBS and rTMS was not statistically different. The findings of a positive effect of TBS vs. sham, and noninferiority of TBS vs. standard HFL rTMS support the continued development of TBS to treat depression.

## Introduction

Newer forms of repetitive transcranial magnetic stimulation (rTMS) have recently been developed, including theta burst stimulation (TBS) for the treatment of major depressive disorder (MDD). TBS sessions commonly last only 3–10 min, compared with conventional rTMS sessions (i.e., 10 Hz stimulation over the left hemisphere [HFL]), which can last up to 40 min. Faster daily treatments with TBS may permit an increase in treatment capacity and the lowering of costs per session^1^. Furthermore, the brevity of daily TBS sessions makes undertaking a course of treatment more convenient and accessible for patients with demanding work schedules or other time commitments.

Several different TBS clinical protocols have shown efficacy for treating MDD: intermittent TBS (iTBS) applied to the left dorsolateral prefrontal cortex (DLPFC); continuous TBS (cTBS) applied to the right DLPFC; and consecutive bilateral cTBS/iTBS applied sequentially to the right and left DLPFC, respectively, in the same session. There have been systematic reviews and meta-analyses of randomized clinical trials (RCTs) in patients with MDD demonstrating that the antidepressant response with different forms of TBS is greater than the response to sham stimulation, with outcomes defined by the Hamilton Rating Scale for Depression (HRSD)^2,3^. However, direct comparisons between TBS and HFL rTMS treatment protocols on clinical outcomes (e.g., categorical response and remission rates) have not been made in a systematic review and meta-analytic manner to date. Additionally, newer RCTs have been published since 2017, so a comprehensive study of the currently available published data is important.

We performed an updated systematic review and meta-analysis of RCTs that evaluated TBS in MDD patients using devices with figure-eight TMS coils. The primary goal was to evaluate all published, peer-reviewed randomized controlled trials comparing TBS (unilateral or bilateral) to HFL rTMS or to sham therapy. We also examined relevant and widely accepted clinical outcomes/endpoints for treating MDD.

## Methods

A systematic search of electronic datasets was undertaken with the intention of including all languages of peer reviewed manuscripts and abstracts. Duplicate published studies were excluded except for cases where additional new data could be extracted from the original study.

The electronic datasets were searched on 10/23/20 and included: PubMed, EBSCO/CINAHL, and the Cochrane Central Register of Controlled Trials (CENTRAL). Searches were undertaken for the dates January 1, 2010 to October 23, 2020. Search terms used were as follows: (((((TMS) AND TBS) AND randomized AND control AND trial AND RCT) AND transcranial magnetic stimulation AND theta burst stimulation) AND major AND depressive) AND disorder. The references of identified RCTs and other systematic reviews and meta-analyses were also hand searched to identify any additional studies. Appendix 1 shows the results of the systematic search.

Peer-reviewed, published manuscripts and abstracts of RCTs were included as long as they met the inclusion criteria: patients >18 years of age, primary diagnosis of MDD, treatment with any form of TBS (intermittent, continuous, or derivations thereof including prolonged or combination protocols), comparison of TBS to HFL rTMS or TBS to sham, treatment >1 week duration; description of clinical endpoints and adverse events/safety. Studies with both treatment naive (no prior MDD treatments) and treatment resistance prior to use of TBS were included. Where multiple publications of the same study existed, maximal data were extracted from these subsequent publications and included as part of the initial publication.

The primary outcome of interest was categorical response as measured by the clinician-rated Hamilton Rating Scale for Depression (HRSD) (≥50% reduction from baseline HRSD total score) at the end of the trial. Secondary outcomes of interest were adverse events, HRSD percent change from baseline score, categorical remission (defined by HRSD 21-item final score <11), baseline-to-endpoint change on the self-report Beck Depression Inventory (BDI), and change on other (either self- or clinician-administered) anxiety or depression severity measures.

### Risk of bias assessment

Two authors evaluated the risk of bias on randomization, treatment allocation, blinding (patient, treatment clinician, and treatment assessor), attrition, selective reporting, and “other” domains. Each domain was graded with regard to risk of bias as low, unclear, or high. Each domain outcome is described in the Cochrane risk of bias tool^4^. A funnel plot to examine publication bias was also generated. A summary of the findings for included studies on risk of bias is included as Appendix 2, along with a list of, and reasons for excluded studies.

### Data synthesis and analysis

Analysis was performed using the statistical software package contained in RevMan software from the Cochrane Collaboration^4^. Two or more studies which measured the same outcome were combined for meta-analysis. Based on assumptions that the true effect size varied amongst the included RCTs and that included trials would have substantial clinical and methodological diversity, a random effects model was employed^5^. For discrete outcomes, the statistical method used was Mantel-Haenszel with the risk-ratio (RR) for effect measures. For continuous outcomes, the inverse variance statistical method was used with standard mean difference (SMD) as the effect measure. Heterogeneity was measured in combined studies using the *I*^*2*^ statistic to evaluate the consistency of findings. If heterogeneity was >50%, a sensitivity analysis was performed to identify the study causing the heterogeneity. If this occurred, the main results were reported with and without heterogeneity, and the sensitivity findings and potential reasons for the heterogeneity were described in the Discussion section. For studies which measured outcomes that were unique from others, the statistics as presented in the published report were included herein. Grading of Recommendations Assessment, Development and Evaluation (GRADE) profiler version 3.6.1 was used to assess the strength and the quality of evidence identified for clinical outcomes^6–8^. Lastly the PRISMA checklist was followed (Appendix 3)^9^.

## Results

### Literature search

**Fig. 1:**
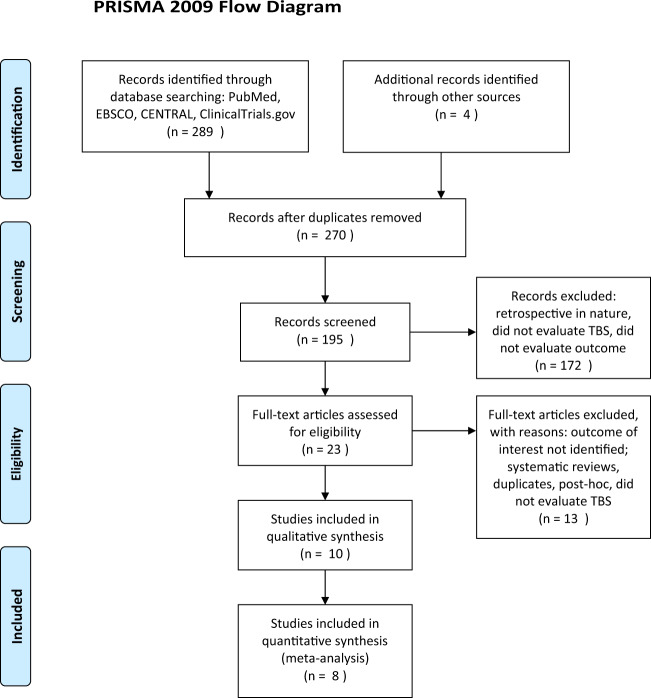
PRISMA flow diagram. Systematic review process for identification of studies included in the analysis.

We screened 195 articles identified upon search from PubMed, EBSCO/CINAHL, and CENTRAL; 23 were retrieved for inclusion assessment. Ultimately 10 articles were included in the meta-analyses^1,10–18^. Of these, nine were single-center trials and one was a multicenter Canadian study^1^. Four studies took place in Germany^12,16–18^, two in Taiwan^14,15^, and one each in Australia^10^, Belgium^13^, Canada^1^, and Israel^11^. There was a total of four studies that employed crossover designs, i.e., from TBS to sham and vice versa^10–13^. In these studies, clinical endpoints were examined prior to crossover to the other therapy. The flow chart in Fig. 1 depicts steps for the selection of studies for qualitative review and meta-analyses. Also, see Appendix 1 for more detail. Table 1 indicates which studies were used for meta-analysis in this study.

**Table 1:** studies used in meta-analysis.

Study	Treatment comparison	Treatment duration	Primary outcome evaluated	Included in Meta-Analysis for these outcomes
Blumberger 2018^1^	TBS stimulation pattern of triplet 50 Hz bursts, repeated at 5 Hz; 2 s on and 8 s off cycle; TBS total duration for 3 min; 9 s (with 600 stimuli per session) vs.; TMS stimulation pattern of 10 Hz frequency repeated at 4 s on and 26 s off cycling vs.; TMS a total duration of 37.5 min (3000 stimuli per session). Intensity of both at 120% of RMT	12 weeks	HRSD scores and response (>50% reduction from baseline)/remission (HRSD score <8)	HRSD response TBS vs. TMS. (Fig. 6) HRSD percent change TBS vs. TMS. (Fig. 8) HRSD remission TBS vs. TMS. (Fig. 9)
Caeyenberghs 2018^10^	TBS stimulation pattern of 50 Hz frequency with a 2 s on 8 s. off cycling. Stimuli were applied at a stimulation intensity of 110% of patient’s RMT. Between 4 daily sessions there was a pause of approximately 15 min each. Each session consisted of 1620 stimuli/session vs.; Sham consisted of a specially designed sham coil identical in form and sound to active coil but did not deliver active stimulation.	1 week	Percent change in HRSD scores from baseline to 1 week out.	BDI TBS vs. sham. (Fig. 10)
Christyakov 2015^11^	TBS stimulation pattern of triple-pulse 50 Hz bursts given at a rate of 5 Hz (2 s between each burst cycling and uninterrupted (3,600 stimuli per session) at an intensity of 100% of patient’s RMT vs.; Sham TBS for a period of 10 days (one session per day). Sham consisted of a specially designed coil which produced identical sounds to active but with no stimulus sensation.	2 weeks	HRSD (21 item) reduction of >50% from baseline (response)	HRSD categorical response TBS vs. sham. (Fig. 5) Adverse events TBS vs. sham. (Fig. 11)
Desmyter 2016^12^	TBS stimulation pattern of 50 Hz frequency with bursts repeated every 2 s and at an intensity of 110% of patient’s RMT. Resulted in 2 s on and 8 s off cycling. Total of 1,620 stimuli per session. Duration of one week (5 sessions per day for a period of 4 days) vs.; Sham coil looked and sounded exactly the same as active coil but without stimuli.	1 week	HRSD (17 item) reduction of >50% from baseline (response)	N/A
Duprat 2016^13^	TBS stimulation pattern of 50 Hz frequency of 54 triplet bursts with a duration of 2 s on and 8 s off cycling. Each session consisted of 1,620 stimulations. Total of 20 TBS sessions spread over 4 days (5 sessions per day; between session pause of 15 min). Stimulation intensity of 110% of patient’s RMT vs.; Sham consisted of identical coil to active placed in same position but without active stimulation.	1 week	HRSD (17 item) reduction of >50% from baseline (response)	HRSD categorical response TBS vs. sham. (Fig. 5)
Li 2014^14^	Group 1: TBS stimulation pattern of 50 Hz frequency with 120 s continuous uninterrupted bursts; with a total of 1,800 stimuli/session for 10 daily sessions over a period of 2 weeks vs; Group 2: TBS stimulation pattern of 50 Hz frequency with 2 s bursts on and 8 s off cycling. Total of 1,800 stimuli/session with10 daily sessions over a period of 2 weeks. Both Group 1 and Group 2 TBS were delivered an intensity of 80% of patient’s RMT vs.; Group 3: Sham TBS bursts randomly assigned as per either Group 1 or 2 with same 1,800 stimuli/session for 10 daily sessions over a period of 2 weeks.	2 weeks	HRSD (17 item) reduction of >50% from baseline (response)	HRSD categorical response TBS vs. sham. (Fig. 5) HRSD percent change TBS vs. sham. (Fig. 7) Adverse events TBS vs. sham. (Fig. 11)
Li 2020^15^	TBS prolonged stimulation pattern of 50 Hz frequency consisting of 1800 stimuli/session: 10 sessions over a 2 week period (1 session per day; 5 sessions per week) vs.; TMS stimulation pattern of 10 Hz frequency consisting of 1600 stimuli/session: 10 sessions over a 2 week period (1 session per day; 5 sessions per week) vs.; Sham [parameters given as prolonged TBS or TMS randomly assigned; using a sham coil]: 10 sessions over a 2 week period (1 session per day; 5 sessions per week).	2 weeks	Percent change in HRSD-17 scores from baseline to 2 weeks out.	HRSD categorical response TBS vs. sham. (Fig. 5) HRSD response rate TBS vs. TMS. (Fig. 6) HRSD percent change TBS vs. sham. HRSD percent change TBS vs. TMS. (Fig. 8) HRSD remission TBS vs. TMS. (Fig. 9)
Mielacher 2019^16^	TBS stimulation consisted of 50 Hz frequency daily sessions [consisting of 2×600 stimuli (1200 stimuli/session) over the left DLPFC] vs.; One active TBS/one sham daily (600 stimuli/session) for a period of 15 sessions over a 3 week period.	3 weeks	Percent change in HRSD-17 scores from baseline to 3 weeks out.	N/A
Plewnia 2014^17^	TBS stimulation pattern of 50 Hz frequency in bursts given every 2 s on and 8 s off cycling of left sided TBS (cycling applied a total of 20 times) followed by right sided continuous (40 s) TBS for a total of 1200 stimuli/session. Intensity of both was 80% of patient’s RMT vs.; Sham was accompanied by similar auditory (clicking noise) and somatosensory (pricking, twitches of temporal muscle) artifact. Both TBS and sham were administered over 6 weeks and 30 total sessions.	6 weeks	MADRS reduction of >50% from baseline (response)	HRSD categorical response TBS vs. sham. (Fig. 5) HRSD percent change TBS vs. sham. (Fig. 7) BDI reduction TBS vs. sham. (Fig. 10) Adverse events TBS vs. sham. (Fig. 11)
Prasser 2014^18^	TMS stimulation consisted of 1 Hz frequency to right DLPFC (1,000 stimuli/session) immediately followed 10 Hz frequency to left DLPFC (1000 stimuli/session). Intensity of TMS performed at 110% of patient’s RMT; TBS stimulation consisted of 50 Hz frequency 1200 pulses/session continuous TBS applied to right DLPFC immediately followed by 1200 stimuli/session of intermittent TBS to left DLPFC. Intensity of TBS performed at 80% of patient’s RMT; sham [consisted of TBS protocol applied with a sham coil]	3 weeks	HRSD-21 change between baseline and 3 weeks (response >50% change; and score <11 points remission)	HRSD categorical response TBS vs. sham. (Fig. 5) HRSD percent change TBS vs. TMS. (Fig. 8) Adverse events TBS vs. sham. (Fig. 11)

*BDI* Beck Depression Inventory, *HRSD* Hamilton Rating Scale for Depression, *RMT* Response Motor Threshold, *TBS* Theta Burst Stimulation, *TMS* Transcranial Magnetic Stimulation.

### Risk of bias assessment

**Fig. 2:**
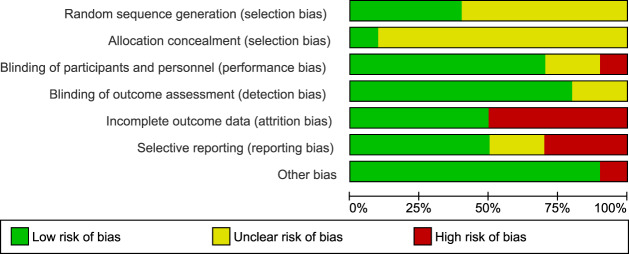
Risk of bias graph. Aggregate evaluation of bias risks for studies included in the analysis.

**Fig. 3:**
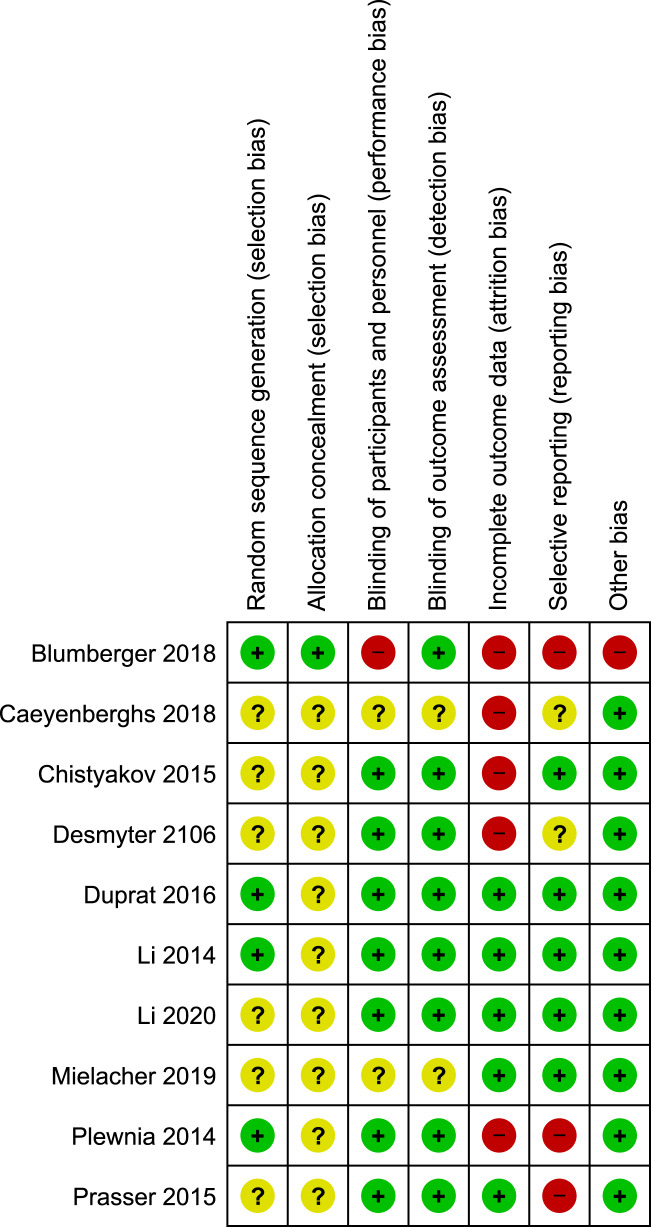
Risk of bias summary. Specific biases as identified for each study included in the analysis.

**Fig. 4:**
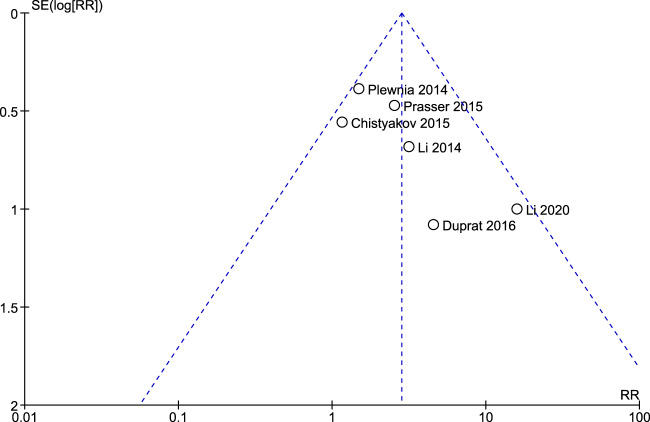
Funnel plot examining publication bias. Intervention effect estimate from individual studies on HRSD response reduction from baseline measured against each study size.

The randomization sequence (i.e., methodology used for random assignment) was identified as having low risk of bias in 4 out of 10 studies^1,13,14,17^. Bias risk in blinding participants and personnel performing the therapy was low in 7 out of 10 studies^11–18^; and bias risk in blinding outcome assessments was low in 8 out of 10 studies^1,11–15,17,18^. In five studies there were high (>10%) attrition rates among enrolled study participants^1,10–12,17^. Three studies selectively reported information, including it in the results section but not identifying it as an endpoint in the methods section^1,17,18^. One study had evidence of potential conflict of interest, e.g. investigators or coauthors disclosed compensation as consultants to the company that funded the trial^1^. Figures 2 and 3 show a risk of bias graph and summary, respectively. Publication bias was also evaluated using a Funnel plot (Fig. 4), with the effect estimate (risk ratio) identified along the horizontal access and standard error identified along the vertical access. The resulting plot resembles a symmetrical inverted funnel thus reflecting an absence of publication bias on response outcome defined as HRSD reduction from baseline of ≥50%.

### Clinical outcomes

#### HRSD response

**Fig. 5:**
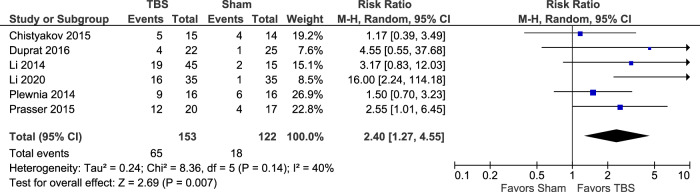
HRSD score ≥50% reduction (categorical response) from baseline TBS vs. sham. Forest plot displaying effect estimates and confidence interveals for both individual studies and overall meta-analysis. Each study is represented by a block at the point estimate of the intervention effect with a horizontal line extending on each side for the confidence interval. The area of the block indicates the weight assigned to that study in the meta-analysis.

**Fig. 6:**
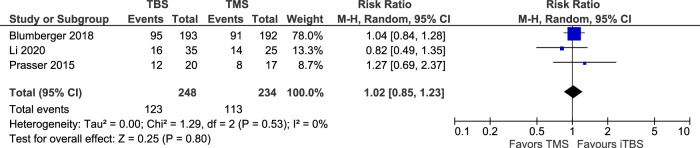
HRSD response rate from baseline TBS vs. TMS. See descriptor in Fig. 5 above.

Six studies evaluated categorical response (≥50% reduction from baseline HRSD total score on the 17- or 21- item scale; one study did not identify the number of HRSD items^17^) at the end of treatment weeks 1–6 in TBS versus sham groups^11,13–15,17,18^. There was a significantly greater number of responders in the TBS than in the sham group (RR = 2.4; 95% CI: 1.27 to 4.55); *P* = 0.007; *I*^*2*^ = 40% (Fig. 5). This outcome was also evaluated using the GRADE profile (Appendix 4). Due to the large effect TBS had on HRSD response, relative to sham, the quality of the evidence was graded as high, indicating high confidence in the estimate of the effect of the intervention. Desmyter et al.^12^ could not be included in the quantitative analysis as per Fig. 5 and in Appendix 4 based on how the data was presented in this crossover design study (sham data could not be extracted). However, it did demonstrate that 39% (18/46) therapy-resistant depressed patients showed ≥50% decrease in the HRSD scores. Comparison of HRSD response rates for those who got active TBS versus active rTMS (3 studies included in the analysis^1,15,18^) produced no statistically significant difference between the 2 therapies when evaluated over a 2–12 week period; RR = 1.02; 95% CI: 0.85 to 1.23); *P* = 0.80; *I*^*2*^ = 0% (Fig. 6).

#### HRSD percent change

**Fig. 7:**
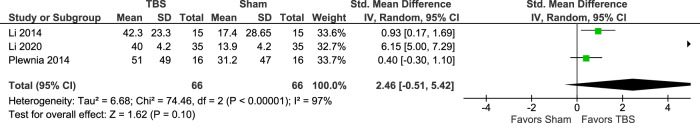
HRSD percent change TBS vs. sham. See descriptor in Fig. 5 above.

**Fig. 8:**
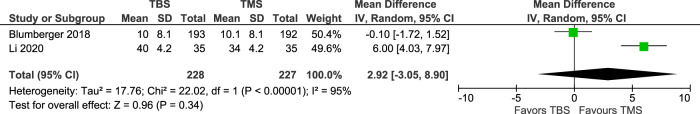
HRSD percent change TBS vs. TMS. See descriptor in Fig. 5 above.

Three studies examined HRSD percent change from baseline scores over 2–6 weeks of treatment^14,15,17^. There was no statistical difference on this endpoint in the analysis of TBS versus sham in a meta-analysis including all three studies (SMD = 2.46; 95% CI: −0.51 to 5.42); *P* = 0.10; *I*^*2*^ = 97% (Fig. 7). Based on the high heterogeneity, a sensitivity analysis was performed with data from the Li et al., 2020^15^ trial excluded (based on the fact that there was no statistically significant difference in HRSD percent change with Li et al. 2020 and that there was a statistically significant difference in the other 2 studies favoring TBS), resulting in a statistically significant HRSD percent change from baseline favoring TBS (SMD = 0.64 95% CI: 0.13 to 1.16); *P* = 0.01; *I*^*2*^ = 0%. (See Discussion section below for elaboration of sensitivity findings for this outcome). When examining the HRSD percent change over a 2–6 week treatment period in analysis of studies comparing TBS vs. rTMS, again no statistically significant difference was found in the outcome (SMD = 2.92; 95% CI: −3.05 to 8.90); *P* = 0.34; *I*^*2*^ = 95% (Fig. 8).

#### HRSD remission

**Fig. 9:**
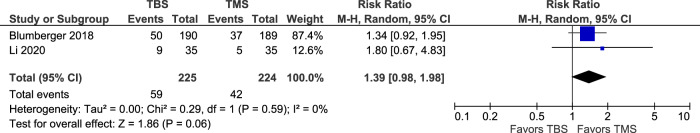
HRSD remission TBS vs. TMS. See descriptor in Fig. 5 above.

Categorical remission, defined as a final score <11 on the HRSD (21 item), was evaluated in one (TBS vs. sham) study^18^. The authors reported no statistically significant difference in remission rates after 11 weeks^18^. Two studies defined remission by an endpoint HRSD (21 item) score ≤7 and; there was no statistically significant difference between TBS and rTMS in the meta-analyses of this outcome evaluated at 2 and 12 weeks^1,15^. However the results, favoring TBS, approached statistical significance (RR = 1.39; 95% CI: 0.98 to 1.98; *P* = 0.06; *I*^*2*^ = 0%) (Fig. 9).

#### Beck depression inventory

**Fig. 10:**
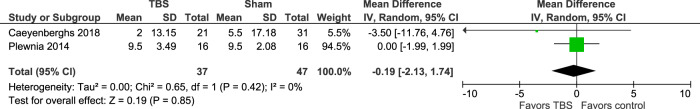
dBDI response from baseline TBS vs. sham. See descriptor in Fig. 5 above.

Absolute reduction in beck depression inventory (BDI) total score from baseline to post-treatment endpoint was evaluated in 2 studies comparing TBS vs. sham after a 1–6 week duration of therapy^10,17^. There was no statistically significant difference between the TBS and sham groups (SMD = −0.19; 95% CI: −2.13 to 1.74); P = 0.85; *I*^*2*^ = 0% (Fig. 10).

#### Other instruments and comparisons evaluated

Outcomes defined by various other assessment instruments and summary metrics have also been used to compare the efficacy of HFL rTMS versus TBS. Results as reported in the individual published reports are summarized in Table 2, below.

**Table 2:** Outcomes of other instruments assessed in single RCTs.

Study	Comparison	Instrument/outcome	Finding
Blumberger 2018^1^	rTMS vs. TBS	IDS-30; response ≥50% from baseline	TBS non-inferior to HFL rTMS
Blumberger 2018^1^	rTMS vs. TBS	QIDS-SR; response ≥50% from baseline	TBS non-inferior to HFL rTMS
Blumberger 2018^1^	rTMS vs. TBS	BSI-A; response ≥50% from baseline	TBS non-inferior to HFL rTMS
Blumberger 2018^1^	rTMS vs. TBS	IDS-30; remission <14	TBS non-inferior to HFL rTMS
Blumberger 2018^1^	rTMS vs. TBS	QIDS-SR; remission <6	TBS non-inferior to rTMS
Desmyter 2014^12^	TBS vs. sham	BSI score	*P* < 0.01 favoring TBS
Plewnia 2014^17^	TBS vs. sham	MARDS ≤ 50% from baseline; response	*P* = 0.048 favoring TBS
Mielacher 2019^16^	Once vs. twice daily TBS	HRSD percent reduction	*P* = 0.043 favoring twice daily TBS

*BSI* Beck Scale for Suicide Ideation (Beck & Steer 1991), *BSI-A* Brief Symptom Inventory-Anxiety subscale (*BSI*; Derogatis & Melisaratos, 1983); *IDS-3* Inventory for Depressive Symptomatology, 30-item (Rush et al. 1996); *MADRS* Montgomery-Åsberg, Depression Rating Scale (Montgomery & Asberg 1979); QIDS-SR = 16 item Quick Inventory of Depressive Symptomatology (Self-Report version) (Rush et al. 2003).

#### Adverse events

**Fig. 11:**
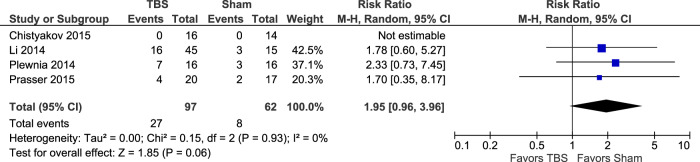
Adverse events TBS vs. sham. See descriptor in Fig. 5 above.

All adverse event data were extrapolated as number of cases per event from the reports of RCTs comparing TBS and sham treatments^11,14,17,18^. The adverse events included both serious (e.g. suicide, hospitalization) and non-serious (e.g. headache, dizziness, nausea, pain) categories. There was no statistically significant difference between the TBS and sham groups when evaluating all adverse events: (RR = 1.95; 95% CI: 0.96–3.96; *P* = 0.06; *I*^*2*^ = 0%)(Fig. 11).

Pooled data from two TBS vs HFL rTMS RCTs also showed no statistically significant difference in adverse events^1,18^ (RR = 0.99; 95% CI: 0.88 to 1.12; *P* = 0.89; *I*^2^ = 0%) (Figure not shown). Self-rated intensity of pain during treatment, a non-serious event, was modestly greater with TBS than HFL rTMS as assessed by a verbal analogue 0-to-10 scale (mean score 3.8 ± 2 vs. 3.4 ± 2; *P* = 0.011) in the one study which collected such data^1^.

Serious adverse events were reported in 4 of the TBS vs. sham RCTs^11,14,17,18^. There was no statistically significant difference between the groups (RR = 5.00; 95% CI:0.26 to 96.59); *P* = 0.29 (Figure not shown). No serious adverse events were reported in two of the TBS vs. HFL rTMS RCTs^1,18^.

## Discussion

In line with prior meta-analyses^2,3,19^, results of the current analysis demonstrated significantly higher response rates in patients with MDD following TBS than sham, as defined by a ≥ 50% reduction on HRSD. Based on the large treatment effect on this categorical response outcome measure as evaluated by GRADE, we found the strength and quality of the evidence for this finding to be high. There was consistency of findings across studies using the same instrument (HRSD) to define response, and consistency across outcomes derived from other instruments, all favoring TBS over sham. This likely bodes well for the robustness of our finding that TBS has a positive clinical effect on MDD.

Where high heterogeneity existed (i.e. in analysis of HRSD percent change comparing TBS versus sham), we found that eliminating one of the studies (Li et al., 2020^15^) resulted in a statistically significant difference favoring TBS. The difference in the eliminated Li 2020 vs. the remaining included studies was that Li 2020 included patients who were trialed on only one pharmacologic medication and then remained medication free for a period of two weeks prior to starting stimulation sessions^15^. The remaining two studies both required resistance to at least two pharmacologic treatment trials and; subjects also remained on stable antidepressant medications prior to and during the course of study treatments^14,17^. Greater variation of clinical effect (measured as percent change in HRSD) may have arisen from inclusion of a population with relatively greater pharmacoresistance or more severe depressive illness in the two studies which were included in the meta-analysis after exclusion of Li et al.^15^. Thus this lower variation in the clinical effect found in Li 2020 (possibly due to a lower resistance to treatment effect with TBS) was different than the wider variation in effect found in both Li 2014^14^ and Plewnia 2014^17^.

Updated data from our study extends the previously reported systematic review and meta-analytic literature comparing TBS to standard HFL rTMS by demonstrating no statistically meaningful differences in response and/or remission outcomes on a number of validated clinician and self-report instruments, including HRSD, IDS-30, QIDS-30, MARDS, and BSI. While TBS efficacy was not statistically different to that of standard rTMS following a standard acute course, we note that this non-statistically significant difference also persisted for 8–12 weeks after treatment^1,15,18^. We also found no statistically significant difference in safety/adverse events between TBS and HFL rTMS therapies based on events reported in published RCTs. In light of the notable advantage of TBS over standard HFL rTMS, i.e. significantly shorter duration of daily stimulation sessions, the noninferiority findings for TBS outcomes hold significant potential for shaping the current care standards for depressed patients. The 3 studies comparing TBS to rTMS included in the meta-analysis represent 482 patients and show a consistency of comparable outcomes between the 2 therapies. The findings, however, should be viewed with caution until more patients are reported on in similar type trials.

TBS appears to be well tolerated. We found the attrition rate (which incorporated the dropout rate) for TBS to be low in five studies, and even though it was somewhat higher (~10–12%) in the other five HFL rTMS studies, these rates are still significantly lower than the average 25% attrition reported for antidepressant medication trials^20^.

While this meta-analysis supports the preliminary efficacy of TBS treatment for MDD, there are questions regarding the optimal approach for administering TBS that remain unanswered. First, while response rates to TBS and other forms of rTMS treatment may be similar in the population of patients with MDD, it remains unclear whether these treatments are equivalent for individual patients. It is possible that there may be a differential response to TBS treatment based upon the degree of resistance to pharmacologic agents, clinical symptoms, or other sources of heterogeneity in the MDD population. For any individual patient, TBS or HFL rTMS may be superior. One recent report indicates that those patients who do not have an early response to HFL rTMS may show greater improvement following the addition of TBS priming (TBS-P) to their treatment regimen^21^. Future studies should examine this heterogeneity in response.

Second, it is important to note several factors that could have affected the findings including: considerable heterogeneity in the TBS protocols and patients reviewed herein, in terms of the number of pulses used; the number of sites stimulated; the fact that some trials allowed for continuation of medication regimens during the trial^1,11,14,17^ and; number of failed trials (1–3) prior to RCT enrollment. Li and colleagues^14,15^ have routinely applied 1800 pulses per session of TBS which may obtain superior clinical outcomes to studies employing 600–1200 pulses/session. As well, some of the RCTs applied stimulation at 80–100% intensity, relative to motor threshold, while others applied it at 110–120%. There are a number of treatment protocols included in this systematic review and meta-analysis that include iTBS either alone or in combination with cTBS and prolonged TBS which are reflective of the attempts to identify an optimal treatment protocol for MDD. It is important to note that there may be complex non-linear effects from increasing the number of TBS pulses. The heterogeneity in these factors could not be controlled for in the present meta-analysis, but should be examined in further studies.

Third, one of the concerns in prior systematic reviews^19^ was a lack of follow-up assessment beyond the end of treatment. While the majority of studies did not have long-term follow-up assessments^10,11,13–16^ after courses of treatment lasting only 2–3 weeks, 2 studies had follow-up assessments and 8–12 weeks after the final stimulation session^1,17^. Blumberger et al.^1^ identified a durability of response to TBS at 12 weeks (measured as a > 50% reduction from baseline on HRSD-17). At 8 weeks post-treatment, Prasser et al.^17^, found a non-significant trend towards a higher response rate (>50% reduction from baseline on HRSD-21) with TBS compared to both rTMS and sham. Thus, there appears to be efficacy in regards to response rates with TBS lasting 2–3 months post-treatment.

Finally, there has been recent interest in the use of more than once-daily TBS treatments to speed treatment response. In one study, twice daily sessions of TBS significantly decreased depressive symptoms 53 ± 23% vs. 36 ± 35% (*P* = 0.043) when evaluated using percent reduction from baseline in the HRSD-17 instrument at the end of 3 weeks of treatment in comparison to once daily treatment (15 sessions)^16^. While this was a small study of 36 patients comparing twice daily (active/active) vs. twice daily (active/sham), it demonstrated the likely need for protocols to evaluate optimization of the use of TBS in treating MDD. A recent open-label study used up to 10 daily sessions of iTBS in psychiatric inpatients and obtained robust response after only five days^22^.

The finding of positive effects of TBS on MDD confirm prior meta-analyses^2,3,19^, expand upon prior findings, and support the growing interest in application of TBS protocols for treating neuropsychiatric disorders such as MDD. This technique may present an opportunity for greater time- and cost-effective care with equivalent safety/adverse events profiles.

## Supplementary information

Appendix 1

Appendix 2

Appendix 3

Appendix 4
