# Supplementary material for: Theta burst stimulation for the acute treatment of major depressive disorder: A systematic review and meta-analysis
Source: Transl Psychiatry. 2021 May 28;11:330. doi: 10.1038/s41398-021-01441-4 (PMC8163818; doi:10.1038/s41398-021-01441-4)
Supplement: Supplementary file 1 — Appendix 1 [file 41398_2021_1441_MOESM1_ESM.pdf]

Appendix 1: Search summary

Search terms used: (((((TMS) AND TBS) AND randomized AND control AND trial AND RCT) AND transcranial magnetic stimulation AND theta burst stimulation) AND major AND depressive) AND disorder.

Database	Date searched	Hits	Duplicates	Screened	Studies	Records obtained
Pubmed, EBSCO	10/23/2020	161	0	161	3	Cayenberghs K. Accelerated intermittent theta burst stimulation in major depression induces decreases in modularity: A connectome analysis. Networks NeuroSci 2018. 3(1): 157-72.
						Bulteau S. et al. Efficacy of intermittent Theta Burst Stimulation (iTBS) and 10-Hz high-frequency repetitive transcranial magnetic stimulation (rTMS) in treatment-resistant unipolar depression: study protocol for a randomised controlled trial 2017. Trials. 18(1):17.doi: 10.1186/s13063-016-1764-8.
						Desmyter, S. Front Hum Neurosci. 2016; Accelerated Intermittent Theta Burst Stimulation for Suicide Risk inTherapy-Resistant Depressed Patients: A Randomized, Sham-Controlled Trial
CENTRAL	10/23/2020	76	7	19	12	Schulze L et al. Effect of antipsychotic pharmacotherapy on clinical outcomes of intermittent theta-burst stimulation for refractory depression Journal of psychopharmacology. 2017, 31(3), 312-319
						Duprat R, et al. Accelerated intermittent theta burst stimulation treatment in medication-resistant major depression: A fast road to remission? Jrl Affect Disord. 2016. 200:6-14.
						Li C-T, et al. Efficacy of prefrontal theta-burst stimulation in refractory depression: a randomized sham controlled study. Brain. 2014. 137:2088-2098.
						Meilacher C. Once daily versus twice daily theta-burst stimulation in the treatment of major depression disorder. Brain stimulation, 2019, 12(2), 397
						Plewnia C. Treatment of major depression with bilateral theta burst stimulation. Journal of affective disorders, 2014, 156, 219-223.

Database	Date searched	Hits	Duplicates	Screened	Studies	Records obtained
						Baeken C, et al. Accelerated theta burst stimulation in treatment resistant major depressive disorder: a fast road to remission? Australian and New Zealand journal of psychiatry, 2016, 50, 41;
						Blumberger D, et al. Intermittent theta-burst versus 10 Hz left dorsolateral prefrontal rTMS for treatment resistant depression: preliminary results from a two-site, randomized, single blind noninferiority trial. Brain stimulation, 2015, 8(2), 329
						Prasser J. Bilateral prefrontal rTMS and theta burst TMS as a add-on treatment for depression: A randomized placebo controlled trial. World journal of biological psychiatry, 2015, 16(1), 57-65
						Li C-T, Chen M-H, Juan C-H, et al. Effects of prefrontal theta-burst stimulation on brain function in treatment-resistant depression: A randomized sham-controlled neuroimaging study. *Brain Stimulation*. 2018. 11:1054-1062.
						Downar J et al. Resting-state functional connectivity to subgenual cingulate cortex differentially predicts treatment response for 10 Hz versus intermittent theta-burst rTMS in major depression. Brain stimulation, 2015, 8(2), 396.
						Li C-T. Antidepressant efficacy of prolonged intermittent theta burst stimulation monotherapy for recurrent depression and comparison of methods for coil positioning: A randomized, double-blind, sham-controlled study. Biological psychiatry. 2020;87:443-450.
						Blumberger DM. Effectiveness of theta burst versus high-frequency repetitive transcranial magnetic stimulation in patients with depression (THREE-D): a randomised non-inferiority trial. Lancet 2018, 391(10131), 1683-1692;
Clinical trials.gov	10/23/2020	52	8	15	4	Vignaud P, Damasceno C, Poulet E, Brunelin J. Impaired Modulation of Corticospinal Excitability in Drug-Free Patients With Major Depressive Disorder: A Theta-Burst Stimulation Study. Front Hum Neurosci. 2019 Feb 26;13:72

Database	Date searched	Hits	Duplicates	Screened	Studies	Records obtained
						Fitzgerald PB, Hoy KE, Elliot D, Susan McQueen RN, Wambeek LE, Daskalakis ZJ. Accelerated repetitive transcranial magnetic stimulation in the treatment of depression. Neuropsychopharmacology. 2018 Jun;43(7):1565-1572. doi: 10.1038/s41386-018-0009-9. Epub 2018 Feb 5
						Mutz J, Vipulananthan V, Carter B, Hurlemann R, Fu CHY, Young AH. Comparative efficacy and acceptability of non-surgical brain stimulation for the acute treatment of major depressive episodes in adults: systematic review and network meta-analysis. BMJ. 2019 Mar 27;364:l1079. doi: 10.1136/bmj.l1079.
						Chung SW, Hill AT, Rogasch NC, Hoy KE, Fitzgerald PB. Use of theta-burst stimulation in changing excitability of motor cortex: A systematic review and meta-analysis. Neurosci Biobehav Rev. 2016 Apr;63:43-64.doi:10.1016/j.neubiorev.2016.01.008. Epub 2016 Feb 3. Review.
Hand search	7/16/2019; BMJ 2019 systematic review	2	0		2	Berlim, M. T., McGirr, A., dos Santos, N. R., Tremblay, S. & Martins, R. Efficacy of theta burst stimulation (TBS) for major depression: an exploratory meta-analysis of randomized and sham-controlled trials. Journal of Psychiatric Research 90, 102-109 (2017).
						Beynel, L. et al. What saccadic eye movements tell us about TMS-induced neuromodulation of the DLPFC and mood changes: a pilot study in bipolar disorders. Frontiers in Integrative Neuroscience 8, 65 (2014).
Hand search	10/24/20; Jr Psych Res. 2017		5		0	
Hand search	10/24/20 - Lancet 2018	1	0		1	Brunoni AR, Chaimani A, Moffa AH, et al. Repetitive transcranial magnetic stimulation for the acute treatment of major depressive episodes: a systematic review with network meta-analysis. JAMA Psychiatry 2017; 74: 143–52.

Database	Date searched	Hits	Duplicates	Screened	Studies	Records obtained
Hand search	10/24/20 - JAMA Psych 2016	1	3		1	Chistyakov AV, Kreinin B, Marmor S, et al. Preliminary assessment of the therapeutic efficacy of continuous theta-burst magnetic stimulation (cTBS) in major depression: a double-blind sham-controlled study. J Affect Disord. 2015;170:225-229.
Sum		293	23	195	23	
Trials used in qualitative review					10	
Trials used in quantitative review					8	
