# Supplementary material for: Theta burst stimulation for the acute treatment of major depressive disorder: A systematic review and meta-analysis
Source: Transl Psychiatry. 2021 May 28;11:330. doi: 10.1038/s41398-021-01441-4 (PMC8163818; doi:10.1038/s41398-021-01441-4)
Supplement: Supplementary file 2 — Appendix 2 [file 41398_2021_1441_MOESM2_ESM.pdf]

Appendix 2: Included and excluded studies

Characteristics of included studies

Blumberger 2018

**Methods**	Randomized, multicenter non-inferiority clinical trial as 3 Canadian university hospitals that took place between September 3, 2013 to October 3, 2016 comparing iTBS to 10 Hz TMS over left dorsolateral prefrontal cortex (LDLPFC); treated once-daily over a period of 12 weeks.
**Participants**	Patients diagnosed with major depressive disorder (MDD) and resistant to 1-3 antidepressant medications trials; HFL TMS group (n=205): average age = 43.2 (12.2); M/F = 42%/58%; iTBS group (n=209): average age = 41.6(10.8); M/F = 39%/61%. Patients excluded if: substance abuse, active suicidal intent, pregnancy, bipolar disorder, psychotic disorder, previous rTMS treatment, prior ECT, cardiac or intracranial implant, unstable medical illness, substantial neurological illness, abnormal serology.
**Interventions**	Triplet 50 Hz bursts, repeated of 5 Hz; 2 s on and 8 s off cycle; TBS total duration for 3 minutes; 9 seconds (600 pulses per session) vs. 10 Hz rTMS with a total duration of 37.5 minutes (3,000 pulses per session). Intensity of both was 120% of resting motor threshold. All participants received 5 sessions per week. Coil position was neuronavigated to LDLPFC stereotactic coordinates for all sessions. All participants continued on stable pharmacotherapy regimens prior to and throughout the study.
**Outcomes**	HRSD scores and response/remission at 1, 4, and 12 weeks; Inventory of Depressive Symptomatology (IDS); Brief Symptom Inventory-Anxiety Scale (BSI-A); Quick Inventory of Depressive Symptomatology (QIDS).
**Notes**	NCT01887782. Investigators have various relationships with industry including rTMS/iTBS companies.

Risk of bias table

Bias	Authors' judgement	Support for judgement
Random sequence generation (selection bias)	Low risk	Randomization tables of a fixed size were made before each site started recruitment with a computer-based algorithm that generated randomly permuted blocks, which were stratified by study site, and groups were balanced regarding degree of medication resistance.
Allocation concealment (selection bias)	Low risk	Patients assigned to treatment arm and then treatment was initiated.
Blinding of participants and personnel (performance bias)	High risk	Participants and treatment technicians were by necessity aware of the treatment condition
Blinding of outcome assessment (detection bias)	Low risk	Staff assessing treatment were masked to treatment allocation.
Incomplete outcome data (attrition bias)	High risk	Of 501 participants that were enrolled, 87 (17%) were ineligible or declined to participate.
Selective reporting (reporting bias)	High risk	Reported on adverse events in results section but did not identify them in the methods section. Also identified serious adverse events in results section but did not define them in methods section.
Other bias	High risk	Several authors have financial consulting relationships with TMS manufacturers.

Caeyenberghs 2018

**Methods**	RCT single center cross-over trial in MDD patients at a single center in Sydney, Australia. Trial dates not reported. Patients were randomly allocated to 2 groups: during first week, one group received active stimulation and the other the sham condition. The treatment

	conditions (sham, active) were reversed during the second week.
**Participants**	Total of 46 adults; mean age = 41.6 (11.7) years; M/F = 14/32; patients resistant to at least one antidepressant pharmacotherapy trial. Exclusion criteria: contraindicated to MRI scan; bipolar or psychotic symptoms; history of epilepsy, cerebral surgery, alcohol dependence, suicidal attempt within 6 months. Antidepressant and antipsychotic medication and mood stabilizer medications discontinued 2 weeks prior to start of blinded treatment.
**Interventions**	Each TBS session consisted of 54 trains of 10 bursts with 3 stimuli. Stimulation of 2 s with a cycling period of 8 s. Stimuli were applied in a 50 Hz frequency and at a stimulation intensity of 110% of patient's resting motor threshold. Between daily sessions there was a pause of approximately 15 minutes each – total of 1,620 pulses per session. TBS applied for a period of 1 week and then crossed over to sham therapy in 2nd week. TBS or sham delivered at 5 sessions per day during 4 days. Sham consisted of a specially designed sham coil identical in form and sound to active coil but did not deliver active stimulation.
**Outcomes**	Depression severity changes using the HRSD administered at 3 time points: baseline, end of week 1 and end of week 2.
**Notes**	NCT01832805. Note: Baeken C, Placebo aiTBS attenuated suicidal ideation and frontopolar cortical perfusion in major depression. Translational Psychiatry. 2019:9:38 was a follow-on study and reported on outcome of suicidal ideation. This outcome was therefore included in Caeyenberghs 2018 as a follow-on analysis.

Risk of bias table

Bias	Authors' judgement	Support for judgement
Random sequence generation (selection bias)	Unclear risk	Unclear as to how patients were randomized to each group.
Allocation concealment (selection bias)	Unclear risk	Unclear as to when treatment allocation occurred and when treatment started

Blinding of participants and personnel (performance bias)	Unclear risk	States study was double blind but unclear as to who was blinded.
Blinding of outcome assessment (detection bias)	Unclear risk	States study was double blind but unclear as to who was blinded.
Incomplete outcome data (attrition bias)	High risk	46 entered the trial and 41 were reported on at end of 2 week trial (11% attrition)
Selective reporting (reporting bias)	Unclear risk	HDRS endpoints identified in methods section were reported on in the results section
Other bias	Low risk	The authors declared no competing interests exist.

Christyakov 2015

**Methods**	RCT single center partial cross-over study, Haifa, Israel, continuous TBS. No date as to when the study was undertaken. Patients were randomized to receive either active or sham. After the 10th session [Phase 1] (performed on consecutive days), the sham randomized patients crossed over the active treatment which consisted of 10 additional daily sessions (Phase 2). Patients who were initially randomized to receive active treatment continued with the same active treatment in Phase 2.
**Participants**	Twenty nine patients with moderate to severe MDD (19 unipolar; 11 bipolar). Patients were hospitalized due to lack of response to prior medication or deterioration of prior condition. Average age: 51.8(14.2); M/F: 11/18; length of current episode (months): 12.5(14.8). Exclusion criteria: suicidal risk, seizure disorder, history of head trauma; uncontrolled medication conditions, pacemaker or metallic implants, drug or alcohol abuse in last 6 mths.
**Interventions**	TBS consisted of triple-pulse 50 Hz bursts given at a rate of 5 Hz (2 s between each burst) (n=15) uninterrupted with 3,600 pulses per session at 100% of patient's RMT vs. sham cTBS (n=14) for a period of 10 days (one session per day). Sham consisted of a specially designed coil which produced identical sounds to active but with no stimulus sensation. . Twenty six patients received concomitant pharmacotherapy and three were medication free throughout trial.

**Outcomes**	HRSD assessment with a (≥50% reduction from baseline HRSD-21 score) defined as a clinical response after 10 sessions.
**Notes**	No conflicts of interest noted. Funding provided by the Niederscachsen Research Foundation.

Risk of bias table

Bias	Authors' judgement	Support for judgement
Random sequence generation (selection bias)	Unclear risk	Randomized but unclear as to randomization scheme.
Allocation concealment (selection bias)	Unclear risk	Unclear as to when patients were assigned to treatment arm and when treatment started.
Blinding of participants and personnel (performance bias)	Low risk	Patients were blinded to treatment. Technician who delivered cTBS was not blinded to treatment and was not involved in the participant's care and assessment
Blinding of outcome assessment (detection bias)	Low risk	HRSD rater blinded to treatment arm.
Incomplete outcome data (attrition bias)	High risk	Three patients dropped out of the study, all from the group that initially received sham cTBS. One patient withdrew after 2 treatment sessions and two additional patients dropped out two days after they were crossed over to active cTBS. (6/29 = 21% drop out rate)
Selective reporting (reporting bias)	Low risk	HRSD outcome as defined in methods section was reported on in the results section.
Other bias	Low risk	No conflicts of interest. Funding provided by the Niederscachsen Research Foundation.

Desmyter 2016

**Methods**	RCT cross over design taking place at a single site in Germany. Unclear as to dates of when study took place. Patients randomized to 2 groups: during first week one group received active stimulation and the other the sham condition. During the second week, each group was crossed over to the other condition.
**Participants**	Patients with MDD were included: Mean age in years: 41.9 (11.77); M/F:18/35; patients were antidepressant medication free at the time of initiation of the trial. Patients also experienced failure of at least one adequate trial of one major class of depressants. Excluded: psychotic symptoms, history of epilepsy, cerebral surgery, pacemaker, having had electroconvulsive therapy (ECT), alcohol dependence, and patients who had committed a suicide attempt within 6 months prior to start of study.
**Interventions**	Each TBS session consisted of 54 trains of 10 bursts of 3 stimuli. Stimuli were applied in a 50 Hz frequency with bursts repeated every 2 s. Resulted in 2 s of stimulation alternated by 8 s rest periods and 1,620 stimuli per session (n=22) vs. sham (n=24) for a period of one week (5 sessions per day for a period of 4 days; Tuesday-Friday) and then crossed over to the other therapy in week 2. Sham consisted of coil that looked identical with a similar noise to active coil without delivering stimulation.
**Outcomes**	Beck scale of suicide ideation (BSI) and HRSD at week 1, 2, 2 weeks after last stimulation, 6 mths after baseline.
**Notes**	NCT01832805. No conflicts of interest noted.

Risk of bias table

Bias	Authors' judgement	Support for judgement
Random sequence generation (selection bias)	Unclear risk	Unclear as to randomization scheme
Allocation concealment (selection bias)	Unclear risk	Unclear as to when patients were randomized to treatment arms and when study was initiated.

Blinding of participants and personnel (performance bias)	Low risk	Patients were blindfolded, wore earplugs and were kept unaware of the type of stimulation. Sham made similar noise to treatment. Clinicians were not blinded.
Blinding of outcome assessment (detection bias)	Low risk	HRSD assessor was blinded to treatment arm. BSI is a self-administered test.
Incomplete outcome data (attrition bias)	High risk	Fifty three patients entered the study, 46 completed it. 7/53 = 13% attrition rate
Selective reporting (reporting bias)	Unclear risk	All outcomes identified in methods section were reported on in results section.
Other bias	Low risk	No conflicts of interest identified.

Duprat 2016

**Methods**	Randomized double blind sham controlled cross-over single center trial taking place at Ghent University, Belgium in patients with major depressive disorder as measured by Hamilton Depression Rating Scale (HDRS) and by self-assessment with Beck Depression Inventory (BDI). Unclear as to dates of trial. Patients were randomized in the first week either 20 real on sham iTBS session (5 sessions/day). During the second week patients were crossed over to the other condition/therapy.
**Participants**	Forty seven patients (33 F/14 M); average age 41.72+/-11.8 years; duration of depressive episode (years): 3.15+/-2.65; antidepressant medication free patients. Patients were at least stage 1 medication resistant (minimum of one unsuccessful treatment trial with serotonin reuptake inhibitors/noradrenaline or serotonin reuptake inhibitors). Exclusion criteria: past history of epilepsy, neurosurgical interventions, pacemaker or metal/magnetic objects in the brain, past electroconvulsive therapy, alcohol dependence, suicide attempts in past 6 mths.
**Interventions**	Each session received 1,620 pulses per session in 54 triplet bursts with train duration of 2 s interval of 8 s off of TBS in 20 TBS sessions spread over 4 days (5 sessions per day; between session pause of 15 minutes)

	at stimulation intensity of 110% of patient's RMT vs. sham. Sham consisted of identical coil to active placed in same position but without active stimulation.
**Outcomes**	Clinical response (≥50% reduction from baseline HRSD-17 score) after 1 week of therapy. Daily self-assessment using BDI.
**Notes**	NCT01832803; Three patients dropped out of initial 50 yielding 47 for assessment. No funding for study and no conflicts of interest reported.

Risk of bias table

Bias	Authors' judgement	Support for judgement
Random sequence generation (selection bias)	Low risk	Randomized via a coin flip to receive either iTBS or sham in first week and then crossed-over to alternative therapy.
Allocation concealment (selection bias)	Unclear risk	Unclear as to when allocation occurred and when trial started
Blinding of participants and personnel (performance bias)	Low risk	Patients were blindfolded, wore earplugs and kept unaware of type of stimulation they received. Personnel administering the therapy were aware.
Blinding of outcome assessment (detection bias)	Low risk	HRSD assessed by a certified psychiatrist blinded to actual treatment of patients.
Incomplete outcome data (attrition bias)	Low risk	Out of 50 entered into the trial 3 dropped out (6% attrition rate)
Selective reporting (reporting bias)	Low risk	All outcomes as identified in the methods section were reported on in the results section.
Other bias	Low risk	None conflicts of interest to report

Li 2014

**Methods**	Randomized controlled trial taking place in Taiwan in patients with MDD at a single center.
**Participants**	60 patients randomly allocated to one of 4 groups: continuous TBS (cTBS), iTBS, cTBS + iTBS, sham; age range 25-64 years of age; M/F = 20/40. Patients were required to have at least 2 failed antidepressant medication treatments. During study patients were required to maintain their original medication regimen. Exclusion criteria: history of psychotic disorder, bipolar disorder; substance abuse; personality disorder; neurologic disorder; brain or cardiac metal implant; pregnancy; major systemic illness
**Interventions**	Continuous TBS (n=15): 120 s train uninterrupted bursts(n=15); 1,800 pulses/session for 10 daily sessions over a period of 2 weeks; Intermittent TBS (n=15): 2 s train bursts repeated every 10 s. 1,800 pulses/session for 10 daily sessions over a period of 2 weeks; cTBS + iTBS (n=15): 1800 pulses/session for 10 daily sessions over a period of 2 weeks. cTBS and iTBS randomly assigned starting from initial iTBS or cTBS. Both cTBS and iTBS at an intensity of 80% of patient's RMT; sham TBS (n=15) bursts given as cTBS or iTBS randomly assigned: 1,800 pulses/session for 10 daily sessions over a period of 2 weeks.
**Outcomes**	HRSD-17 response (≥50% reduction from baseline) after 2 weeks. Adverse events; Standard uptake values (SUV) of cerebral glucose metabolism, at rest, both before and after 10 daily treatment sessions.
**Notes**	No conflicts to report. Li 2018 study data (Li C-T et al. Effects of prefrontal theta-burst stimulation on brain function in treatment-resistant depression: A randomized sham-controlled neuroimaging study. Brain Stimulation 2018;11:1054-1062) duplicate of Li 2014 and data extraction incorporated into Li 2014 study.

Risk of bias table

Bias	Authors' judgement	Support for judgement

Random sequence generation (selection bias)	Low risk	Computerized random number generator with the block randomization method (block size of 8) used.
Allocation concealment (selection bias)	Unclear risk	Unclear as to when treatment allocation occurred and when treatment began.
Blinding of participants and personnel (performance bias)	Low risk	We questioned all patients about group assignment at week 2; none of the recruited patients admitted they knew for sure which group they had been assigned to. Clinicians treating patients knew which arm they were allocated to.
Blinding of outcome assessment (detection bias)	Low risk	All efficacy outcomes were assessed by blinded study personnel (raters) who were not permitted access to treatment sessions.
Incomplete outcome data (attrition bias)	Low risk	All patients who entered trial completed it.
Selective reporting (reporting bias)	Low risk	All outcomes identified in methods section were reported on in results section.
Other bias	Low risk	None of the authors in this study had any conflict of interest to declare.

Li 2020

**Methods**	RCT with MDD patients who have failed at least one adequate antidepressant treatment of current episode. Study undertaken at a single center in Taiwan.
**Participants**	Patients with MDD who had failed one antidepressant treatment and who were antidepressant free for at least one week. Three arms of trial: prolonged iTBS: average age: 47.1(14.2); M/F:12/23; rTMS: average age: 47.1(13.8); M/F: 11/24; Sham: average age: 47.1(12.4); M/F:11/24. Exclusion criteria: psychotic disorders, bipolar disorders, organic mental disorders, prevailing strong suicidal risk.
**Interventions**	Prolonged TBS [1,800 pulses/session] (N=35): 10 sessions over a 2 week period (1 session per day; 5

	sessions per week); rTMS [1,600 pulses/session] (N=35): 10 sessions over a 2 week period (1 session per day; 5 sessions per week); Sham [parameters given as prolonged TBS or rTMS randomly assigned; using a sham coil] (N=35): 10 sessions over a 2 week period (1 session per day; 5 sessions per week)
**Outcomes**	Percent change, response (≥50% reduction compared to baseline, remission rate in HRSD-17 after 2 weeks intervention; safety defined as spontaneous adverse events; degree of refractoriness using Maudsley Staging Method (MSM)
**Notes**	Registered under: UMIN000020892 (University Hospital Medical Information Network Clinical Trials Registry).

Risk of bias table

Bias	Authors' judgement	Support for judgement
Random sequence generation (selection bias)	Unclear risk	RCT - however randomization scheme not identified
Allocation concealment (selection bias)	Unclear risk	Unclear as to when patients were allocated to treatment and when treatment was initiated
Blinding of participants and personnel (performance bias)	Low risk	Patients blinded with a sham coil. All participants were asked about treatment experiences after 2 weeks of treatment and none were confident about their group assignments. Treatment clinicians not blinded
Blinding of outcome assessment (detection bias)	Low risk	Blinded study personnel raters.
Incomplete outcome data (attrition bias)	Low risk	All patients who entered study completed it.
Selective reporting (reporting bias)	Low risk	All outcomes as identified in methods section were reported on in results section.
Other bias	Low risk	Authors reported no financial interests or conflicts of interest.

Mielacher 2019

**Methods**	RCT single center trial taking place in Bonn, Germany. Unclear as to dates study took place.
**Participants**	Thirty six inpatients with MDD; average age: 48(12); M/F: 16/20;
**Interventions**	Two active TBS daily sessions [consisting of 2 X 600 pulses over the left DLPFC] (n=17) vs. One active/one sham daily (n=19) for a period of 15 sessions over a 3 week period
**Outcomes**	HRSD-17 percent reduction from baseline to3 weeks out and side effects.
**Notes**	Abstract only. No competing interests identified.

Risk of bias table

Bias	Authors' judgement	Support for judgement
Random sequence generation (selection bias)	Unclear risk	Unclear as to how patients were randomized
Allocation concealment (selection bias)	Unclear risk	Unclear as to when patients were allocated to treatment arms and when trial was initiated.
Blinding of participants and personnel (performance bias)	Unclear risk	Study stated was double blind but unclear as to who was blinded.
Blinding of outcome assessment (detection bias)	Unclear risk	Study stated was double blind but unclear as to who was blinded.
Incomplete outcome data (attrition bias)	Low risk	All patients entered into trial completed trial.
Selective reporting (reporting bias)	Low risk	Outcomes identified in methods section were reported on in results section
Other bias	Low risk	No competing interests identified.

Plewnia 2014

**Methods**	Randomized controlled trial. Patients recruited from dept Psychiatry at University Hospital Tubingen, Germany and over a period of 6 weeks. Unclear as to the dates the trial took place.
**Participants**	32 patients with major depression enrolled; 12 M/20 F; Average age TBS: 46.9(13.2); Sham: 49(13.6); 22 patients treatment resistant to pharmacologic treatment (two different antidepressant medications over a period of 4 weeks each). Patients were on antidepressant medication for a period of at least 2 weeks and remained so until the end of the trial. Exclusion criteria: seizures, neurological disorders, previous brain injury; magnetic implants in brain, cardiac pacemaker, psychotic symptoms, substance abuse, pregnancy, deep brain stimulation.
**Interventions**	Each stimulation session consisted of two trains of 600 pulses applied (total 1,200 pulses/session) in bursts of 3 pulses at 50 Hz given every 2 sof left sided iTBS plus right sided followed by right sided cTBS. Left sided stimulation with intermittent TBS was applied 20 times for 2 seconds every 10 seconds. Right sided stimulation with continuous TBS was applied continuously for 40 seconds. Intensity of both was 80% of patient's RMT. Sham was accompanied by similar auditory (clicking noise) and somatosensory (pricking, twitches of temporal muscle) artifact. . Both TBS and sham were administered over 6 weeks and 30 total sessions.
**Outcomes**	Montgomery-Asberg Depression Rating Scale (MADRS); HRSD (number items not listed) and BDI reduction of ≥50% compared to baseline at end of treatment (response)
**Notes**	NCT01153199; no conflicts of interest noted

Risk of bias table

Bias	Authors' judgement	Support for judgement
Random sequence generation (selection bias)	Low risk	Patients randomized using a single computer generated random sequence number.

Allocation concealment (selection bias)	Unclear risk	Unclear as to when patients were allocated and when trial was initiated
Blinding of participants and personnel (performance bias)	Low risk	Those administering test were aware of treatment allocation. Patients were blind to treatment
Blinding of outcome assessment (detection bias)	Low risk	Rates of depression scales were blind to treatment
Incomplete outcome data (attrition bias)	High risk	Of 32 that entered trial; 12 were excluded due to: patient decision, worsening of depression, safety reasons.
Selective reporting (reporting bias)	High risk	All outcomes identified in methods section were reported on in results section.
Other bias	Low risk	No conflicts of interest noted.

Prasser 2015

**Methods**	RCT patients with MDD recruited April 2010 - September 2011, single center University Regensberg, Germany.
**Participants**	Fifty four patients; rTMS (n=17): age years: 50.4(9.9); M/F:9/8; TBS (n=20): age years: 48.2(10.9); M/F:10/10; sham (n=17): age years: 42.6(12.4); M/F:8/9. Exclusion criteria: baseline Hamilton Rating Scale for Depression (HAMD) <18 points; cardiac pacemaker or other implanted electrical device, history of epilepsy or brain damage, alcohol or substance abuse, unstable medical conditions, concurrent medication, inability to comply with study procedure, insufficient knowledge German language. Unknown number of failed trials of therapies prior to TMS/TBS therapy. Patients were medication free at the start of the trial and throughout til the end of the trial.
**Interventions**	rTMS [consisted of 1,000 pulses/session at 1 Hz to right DLPFC immediately followed by 1,000 pulses/session at 10 Hz to left DLPFC (n=17). Intensity of rTMS performed at 110% of patient's RMT; TBS [consisted of 1,200 pulses/session continuous TBS applied to right DLPFC immediately followed by 1,200 pulses/session of intermittent TBS to left DLPFC] (n=20). Intensity of TBS performed at 80% of patient's RMT; sham

	[consisted of TBS protocol applied with a sham coil] (n=17) for a period of 3 weeks (5 sessions per week)
**Outcomes**	HRSD-21 change between baseline and 3 weeks (response); HRSD change over the course of the trial; responder and remitter rates at end of treatment and at end of follow-up period (11 weeks); changes in Beck Depression Inventory (BDI); Clinical Global Impression scale (CGI); Global Assessment of Functioning scale (GAF) over course of trial.
**Notes**	NCT01240083. No conflicts of interest, financial or otherwise.

Risk of bias table

Bias	Authors' judgement	Support for judgement
Random sequence generation (selection bias)	Unclear risk	Patients randomized but unclear as to randomization scheme
Allocation concealment (selection bias)	Unclear risk	Unclear as to when patients allocated to treatment arm and when treatment started.
Blinding of participants and personnel (performance bias)	Low risk	Blinding assessed at week 11 of patients and revealed patients were not able to identify treatment group with a significant accuracy above chance level. Treating clinicians were not blinded.
Blinding of outcome assessment (detection bias)	Low risk	Blinding assessed at week 11 of rating physicians and revealed physicians were not able to identify treatment group with a significant accuracy above chance level
Incomplete outcome data (attrition bias)	Low risk	Fifty six patients were entered into trial. Two of the 56 withdrew their consent (one of sham group after first treatment session; one of rTMS group after randomization but before first treatment)
Selective reporting (reporting bias)	High risk	Did not address number of remitters as identified in methods section in the results section. As well reported on adverse events in results section but

		did not define them in the methods section.
Other bias	Low risk	No conflicts of interest were noted.

Characteristics of excluded studies

Baeken 2017[1]

Reason for exclusion Duplicate of Duprat 2016

Baeken 2019[2]

Reason for exclusion RCT but outcome of interest not evaluated - only suicidal ideation

Berlim 2017[3]

Reason for exclusion Systematic review and meta-analysis of RCTs. Used for additional data extraction.

Brunoni 2016[4]

Reason for exclusion Systematic review and meta-analysis of RCTs. Used for additional data extraction.

Fitzgerald 2018[5]

Reason for exclusion Evaluated accelerated TMS (and not TBS) vs. standard TMS in a RCT

Li 2018[6]

Reason for exclusion Duplicate of Li 2014. Maximal data extracted from Li 2018 and included in Li 2014 analysis.

Mendlowitz 2019[7]

Reason for exclusion Post hoc cost analysis of Blumberger 2018 trial. Cost analysis was not a pre-determined endpoint. As well

direct costs were estimated after the fact and not captured during the clinical trial.

Mutz 2019[8]

**Reason for exclusion**	Systematic review and meta-analysis of RCTs. Used for additional data extraction.

References to excluded studies:

---

[1] Baeken C, Duprat R, Wu G-R, De Raedt R, van Heeringen K. Subgenual anterior cingulate-medial orbitofrontal functional connectivity in medication-resistant major depression: A neurobiological marker for accelerated intermittent theta burst stimulation treatment? *Biol Psych*. 2917. 2:556-565.

[2] Baeken C. Wu G-R, van Heeringen K. Placebo aiTBS attenuated suicidal ideation and frontopolar cortical perfusion in major depression. *Trans Psych*. 2019. 9:38/

[3] Berlim MT, McGirr A, Rodrigues dos Santos N, Tremblay S, Martins R. Efficacy of theta burst stimulation (TBS) for major depression: An exploratory meta-analysis of randomized and sham-controlled trials. *Jrl Psych Res*. 2017. 90:102-109.

[4] Brunoni AR, Chaimani A, Moffa AH, et al. Repetitive transcranial stimulation for the acute treatment of major depressive episodes. A systematic review with network meta-analysis. *JAMA Psych*. 2017. 74(2):143-153.

[5] Fitzgerald PB, Hoy KE, Elliot D, McQueen RNS, Wambeek LE, Daskalakis ZJ. Accelerated repetitive transcranial magnetic stimulation in the treatment of depression. *Neuopsychopharmacology*. 2018. 43:1565-1572.

[6] Li C-T, Chen M-H, Juan C-H, et al. Effects of prefrontal theta-burst stimulation on brain function in treatment-resistant depression: A randomized sham-controlled neuroimaging study. *Brain Stimulation*. 2018. 11:1054-1062.

[7] Mendlowitz AB, Shanbour A, Downar J, et al. Implementation of intermittent theta burst stimulation compared to conventional repetitive transcranial magnetic stimulation in patients with treatment resistant depression: A cost analysis. *PLOS ONE*. 2019. 14(9): e0222546.

[8] Mutz J, Vipulananthan V, Carter B, Hurlemann R, Fu CHY, Young AH. Comparative efficacy and acceptability of non-surgical brain stimulation for the acute treatment of major depressive episodes in adults: a systematic review and network meta-analysis. *BMJ*. 2019. 364:l1079.
