# Supplementary material for: Theta burst stimulation for the acute treatment of major depressive disorder: A systematic review and meta-analysis
Source: Transl Psychiatry. 2021 May 28;11:330. doi: 10.1038/s41398-021-01441-4 (PMC8163818; doi:10.1038/s41398-021-01441-4)
Supplement: Supplementary file 4 — Appendix 4 [file 41398_2021_1441_MOESM4_ESM.pdf]

Appendix 4: GRADE profile

iTBS compared to sham for HRDS >50% response

Bibliography: Christyakov AV, Kreinin B, Marmor S, Kaplan B, Khatib A, Darawsheh N, Koren D, Zaaroor M, Klein E. Preliminary assessment of the therapeutic efficacy of continuous theta-burst magnetic stimulation (cTBS) in major depression: A double-blind sham-controlled trial. Jrl Affect Disor. 2015;170:225-229. Duprat R, Desmyter S, Rudi DR, van Heeringen K, Van den Abbeele D, Tandt H, Bakicv J, Pourtois G, Dedoncker J, Vervaet M, Van Autreve S, Lemmens DMD, Baeken C. Accelerated intermittent theta burst stimulation treatment in medication-resistant major depression: A fast road to remission? Jrl Affect Disord. 2016. 200:6-14. Li C-T, Chen M-H, Juan C-H, Juang H-H, Chen L-F, Hsieh J-C, Tu P-C, Bai Y-M, Tsai S-J, Lee Y-C, Su T-P. Efficacy of prefrontal theta-burst stimulation in refractory depression: a randomized sham controlled study. Brain. 2014. 137:2088-2098. Li C-T, Cheng C-M, Chen M-H, Juan C-H, Tu P-C, Bai Y-M, Jeng J-S, Lin W-C, Tsai S-J, Su T-P. Antidepressant efficacy of prolonged intermittent theta burst stimulation monotherapy for recurrent depression and comparison of methods for coil positioning: A randomized, double blind, sham-controlled trial. Biol Psych. 2020. 87:443-450. Plewnia C, Pasqualetti P, Große, Schlipf S, Wasserka B, Zwissler B, Fallgatter A. Treatment of major depression with bilateral theta burst stimulation: A randomized controlled pilot trial. Jr. Affect Disord. 2014;156:219-223. Prasser J, Schecklmann M, Poeppl TB, FFrank E, Kruezer PM, Hajak G, Rupprecht R, Landgrebe M. Langguth B. Bilateral prefrontal rTMS and theta burst TMS as an add-on treatment for depression: A randomized placebo controlled trial. World Jr Biol Psych. 2015. 16:57-65.

Outcomes	No of Participants (studies) Follow up	Quality of the evidence (GRADE)	Relative effect (95% CI)	Anticipated absolute effects	
				Risk with Sham	Risk difference with ITBS (95% CI)
**HRSD response >50%** Hamilton Rating Scale for Depression	275 (6 studies) 2 weeks	⊕⊕⊕⊕ **HIGH**[1,2] due to large effect	**RR 2.4** (1.27 to 4.55)	**Study population**	
				**148 per 1000**	**207 more per 1000** (from 40 more to 524 more)
				**Low**	
				**50 per 1000**	**70 more per 1000** (from 13 more to 178 more)
				**High**	
				**600 per 1000**	**840 more per 1000** (from 162 more to 1000 more)

*The basis for the **assumed risk** (e.g. the median control group risk across studies) is provided in footnotes. The **corresponding risk** (and its 95% confidence interval) is based on the assumed risk in the comparison group and the **relative effect** of the intervention (and its 95% CI).

CI: Confidence interval; **RR:** Risk ratio;

GRADE Working Group grades of evidence
High quality: Further research is very unlikely to change our confidence in the estimate of effect.
Moderate quality: Further research is likely to have an important impact on our confidence in the estimate of effect and may change the estimate.
Low quality: Further research is very likely to have an important impact on our confidence in the estimate of effect and is likely to change the estimate.
Very low quality: We are very uncertain about the estimate.

[1] While there were few patients included in the studies, there were a large number of events relative to the patients included in the RCT
[2] There was a large effect RR > 2
